# Physicochemical Properties of Poly-vinyl Polymers and Their Influence on Ketoprofen Amorphous Solid Dispersion Performance: A Polymer Selection Case Study

**DOI:** 10.3390/pharmaceutics12050433

**Published:** 2020-05-08

**Authors:** Emer Browne, Zelalem A. Worku, Anne Marie Healy

**Affiliations:** SSPC The SFI Research Centre for Pharmaceuticals, School of Pharmacy and Pharmaceutical Sciences, Trinity College Dublin, Dublin 2, Ireland; brownee3@tcd.ie (E.B.); workuz@tcd.ie (Z.A.W.)

**Keywords:** amorphous solid dispersion, polymer selection, ketoprofen, relative humidity induced glass transition, supersaturation

## Abstract

When developing an amorphous solid dispersion (ASD), a prudent choice of polymer is critical to several aspects of ASD performance including: processability, solid state stability and dissolution rate. However, there is little guidance available to formulators to aid judicious polymer selection and a “trial and error” approach is often taken. This study aims to facilitate rational polymer selection and formulation design by generating ASDs using a range of poly-vinyl polymers and ketoprofen as a model active pharmaceutical ingredient (API) and evaluating several aspects of their performance. The molecular weight of the polymer and the ratio of vinyl pyrrolidone to vinyl acetate in the polymer were found to influence the relative humidity at which the relative humidity induced glass transition occurred, as well as the extent of ketoprofen supersaturation achieved during dynamic solubility testing. Interestingly, ASD tablets containing polymers with the vinyl pyrrolidone functional group exhibited higher tensile strengths than those without. This points towards the binder functionality of vinyl pyrrolidone. In conclusion, the physicochemical properties of poly-vinyl polymers greatly influence ketoprofen ASD performance and due regard should be paid to these properties in order to develop an ASD with the desired attributes.

## 1. Introduction

The poor aqueous solubility of active pharmaceutical ingredients (APIs) is a recurring issue that formulation scientists have faced over the last thirty years due to the physicochemical profile of pipeline APIs [1,2]. A popular strategy to improve the aqueous solubility, rate and extent of dissolution of these APIs involves converting the API into the amorphous form [3].

However, the physical instability of the amorphous form of an API over a pharmaceutically relevant time frame may present a major risk to this strategy. The most popular approach to mitigate this risk is to develop an amorphous solid dispersion (ASD) i.e., to disperse the amorphous API in a carrier (often a polymer) which prevents crystallization of the API. The method of generating an ASD i.e., the manufacturing method can influence the stability of ASD via its influence on the degree of drug–polymer mixing [4] and the particle size and porosity of the resultant product [5]. The choice of polymer to use in ASD development is also critical as it may influence the dissolution rate of the API [6], the propensity towards supersaturation [7] and the processability of the ASD [8]. Clearly, the rational choice of polymer is a vital step in the successful development of an ASD. However, polymer selection is often based on a “trial and error” approach and a systematic guide for polymer selection based on their physicochemical properties is lacking. Physicochemical properties of polymers which should be considered during formulation development include; glass transition temperature, aqueous solubility, molecular weight, hygroscopicity and potential for stabilization via intermolecular interactions between the polymer and the API. 

The role of the molecular weight of the polymer in preventing the recrystallization of an API in an ASD is an important factor to consider. For a given polymer, as the molecular weight increases, the glass transition temperature of the polymer increases. This increase in glass transition temperature of the polymer will, if mixed with the API at the molecular level, lead to an increase in the glass transition of the ASD, which may increase the stability of the ASD during storage. This has been borne out in the literature to varying degrees. Increasing polyvinyl pyrrolidone (PVP) molecular weight was shown to reduce nucleation and crystal growth of piroxicam [9], while in a separate study, it was shown to have little effect on the ability of amorphous indomethacin to crystallize from an ASD [10]. The molecular weight of the polymer also plays a critical role in the solubility and dissolution rate of the API in an ASD in aqueous media. Selecting a polymer of high molecular weight may retard drug dissolution rate from the ASD due to an increase in the viscosity of the diffusion layer [11]. This may be desirable or not depending on the target drug release profile. In the case of ketoprofen, immediate release ASDs have been formulated through selection of low molecular weight hydroxypropyl cellulose (HPC) [8]. In the same study, the authors found that, in compression studies, the lower molecular weight HPC resulted in an ASD which had less elastic recovery and higher plasticity than the equivalent higher molecular weight HPC formulation. This meant that the lower molecular weight HPC formulation was more suitable for processing by direct compression.

While the influence of polymer molecular weight is clearly significant in ASD formulation development, it has been shown to have virtually no effect on the solubility of the API in the polymer [12]. Conversely, the ratio of co-polymer substituents was found to have a significant influence on celecoxib solubility in polyvinyl pyrrolidone vinyl acetate (PVPVA) systems [13]. As the proportion of vinyl-pyrrolidone to vinyl-acetate increased, the solubility of indomethacin in the polymer increased. 

Co-polymer substitution ratio also affects polymer hygroscopicity. The tendency of an ASD to sorb moisture is critical to its propensity to undergo moisture induced phase separation (MIPS) [14], which may precede drug crystallization. The PVPVA co-polymer (which is typically present in commercial ASD preparations as a 6:4 VP:VA ratio) is less hygroscopic than PVP and has been demonstrated to sorb less moisture than PVP when formulated as an ASD [14]. This is likely due to the complete insolubility of vinyl acetate in water [15]. This means that water is less likely to be sorbed due to repulsive effects. While the vinyl acetate functional group may be useful in terms of protection of the ASD from moisture during storage, it may limit the dissolution rate of the amorphous drug in aqueous media. The ability to identify the optimal ratio of VP:VA where dissolution rate is high while moisture sorption is low is therefore important.

The prudent choice of a stabilizing polymer should also consider the propensity of a polymer to form intermolecular interactions with the API. The formation of intermolecular interactions in an ASD can reduce the molecular mobility of the API and prevent crystallization. This has been borne out experimentally in ASDs composed of amorphous nifedipine and one of PVP, hydroxypropylmethylcellulose acetate succinate (HPMCAS) or polyacrylic acid (PAA) [16]. Of the three polymers, PVP was found to have the strongest hydrogen bonding with nifedipine which resulted in a 65-fold increase in relaxation time and the highest resistance to nifedipine recrystallizing. In an older study, PVP was found to hydrogen bond to probucol allowing ASD formation, while polyethylene oxide (PEO) and PAA did not, resulting in probucol being present in a polymorphic crystalline state [17]. Interestingly, hydrogen bonding between polymer and drug may be disrupted during the direct compression process leading to phase separation [18], particularly for metastable formulations. Worku et al. described phase separation of the hydrogen bonded naproxen–PVP ASDs upon compression, while no such phase separation was observed for equivalent naproxen–PVPVA ASDs where less hydrogen bonding is present [19].

Bearing all the above factors in mind, the aim of the current work is to examine the influence of physicochemical properties of a range of polyvinyl-based polymers on the stability and performance of an ASD in order to guide rational polymer selection. Ketoprofen was used as a model API. Polyvinyl alcohol, polyvinyl pyrrolidone (of differing molecular weights), polyvinyl pyrrolidone vinyl acetate in a 7:3 VP:VA ratio and a 3:7 VP:VA ratio, polyvinyl acetate and polyvinyl acetate phthalate were used to probe these effects. Differences relating to solid state characteristics, physical stability, solubility and compression behavior were determined for composite systems of the API and the different poly-vinyl polymers investigated.

## 2. Materials and Methods 

### 2.1. Materials 

**Figure 1:**
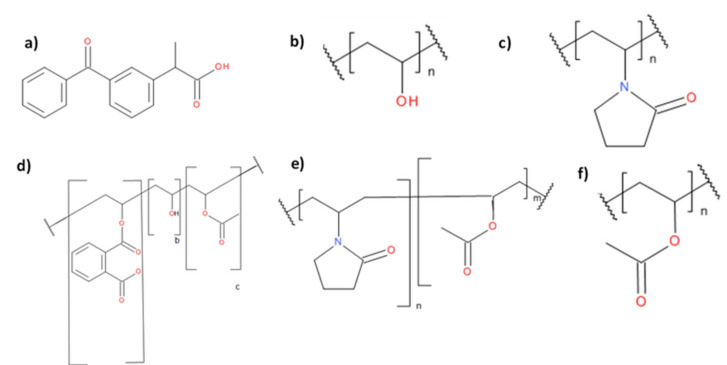
Molecular structures of (**a**) ketoprofen, (**b**) polyvinyl alcohol (PVAlcohol), (**c**) polyvinyl pyrrolidone (PVP), (**d**) polyvinyl acetate phthalate (PVAP), (**e**) polyvinyl pyrrolidone vinyl acetate (VP:VA), (**f**) polyvinyl acetate (PVAcetate).

R,S-ketoprofen (98% purity) was purchased from Sigma Aldrich (St. Louis, MO, USA). Table 1 details the polymers used in this work as well as their sources. For brevity, henceforth each polymer will be referred as the short code highlighted in bold in Table 1. The molecular structures of the polymers selected and ketoprofen are shown in Figure 1. Where used, ethanol was of reagent grade and was sourced from T.E. Laboratories (Tullow, Ireland). Microcrystalline cellulose (MCC) (Avicel PH 102) was purchased from FMC Biopolymer (Brussels, Belgium). 

### 2.2. Methods

#### 2.2.1. Preparation of Solid Samples of Polymeric Solutions via Spray Drying

As VP:VA 3:7 and VP:VA 7:3 were supplied from the manufacturer as 50% *w*/*w* ethanolic solutions, it was necessary to process these solutions via spray drying to obtain a fine powder suitable for ASD manufacture. The solutions as supplied were diluted with ethanol to a 2% *w*/*v* solution and spray dried using a B-290 Mini Spray Dryer (Büchi, Flawil, Switzerland) equipped with a high efficiency cyclone configured in the open mode with an inlet temperature of 78 °C. The solution feed rate was ca. 9–10 mL/min, the drying air flow was ca. 35 m^3^/hr and the atomization air flow was ca. 667 normliters/hr at standard temperature and pressure. The outlet temperature fluctuated between 45 and 48 °C.

#### 2.2.2. Cryo-Milling

Cryo-milling was carried out using a Cryogenic Mixer Mill CryoMill (Retsch, Haan, Germany) attached to an auto-filling liquid nitrogen dewar (Retsch, Haan, Germany). A stainless steel cryo-mill chamber of 25 mL capacity filled with three stainless steel balls of 12 mm diameter were used. An automatic precooling step was applied before any milling was commenced. This consisted of the mill operating at a frequency of 5 Hz while liquid nitrogen circulated around the stainless-steel chamber until cryotemperature was reached, which was automatically detected via a sensor. The material was milled for three cycles, where each cycle consisted of a grinding step at a frequency of 30 Hz for 5 minutes followed by an intermediate cooling step at a frequency of 5 Hz for 2 minutes. Cryotemperature was maintained throughout milling via liquid nitrogen circulating around the milling chamber. As PVAlcohol and PVAcetate were supplied as large grains/ beads (approx. 3 mm and 5 mm in length respectively), it was necessary to process these using a cryo-mill prior to physically mixing these with ketoprofen. Then, 1 g of these materials as supplied were placed in the cryo-mill chamber and milled as described above. 

#### 2.2.3. Preparation of ASDs

To generate ASDs, a physical mixture of ketoprofen and polymer in a 20:80 *w*/*w* ratio were melt-quenched in an aluminum weighing boat by placing on a PC-400D hot plate (Corning, New York, NY, USA) at 120 °C for 10 min before being quenched in liquid nitrogen and 500 mg of this material was then cryo-milled as outlined above to obtain a powder. This was necessary as some of the systems formed glassy films upon melt quenching, which were unsuitable for dissolution and stability testing. 

#### 2.2.4. Solid-State Characterization 

##### Powder X-ray Diffraction (pXRD)

Samples were analyzed using a Miniflex II X-ray diffractometer (Rigaku, Neu-Isenburg, Germany) with Ni-filtered Cu Kα radiation (1.54 Å). The tube voltage and tube current used were 30 kV and 25 mA respectively. The samples were analyzed on a silicon zero-background sample holder in the reflection mode. Diffraction patterns were collected for 2θ ranging from 5° to 40° at a step scan rate of 0.05° per second.

##### Modulated Differential Scanning Calorimetry (mDSC)

The glass transition onset temperatures of polymer–ketoprofen systems were determined by creating melt-quenched systems in situ in a TA Q200 DSC (TA Instruments, Elstree, U.K.) equipped with an RCS-90 refrigerated cooling system (TA Instruments, Elstree, U.K.). The temperature was calibrated with indium and tin and further validated with indium. The enthalpy and the specific heat capacity were calibrated and verified using indium and sapphire, respectively and the furnace was purged with nitrogen gas at a flow rate of 50 mL/min. Samples were first mixed in an agate pestle and mortar (80:20 *w*/*w* polymer: ketoprofen) and accurately weighed (2–6 mg) into standard aluminum pans with a capacity of 20 μL. Pans were sealed with standard aluminum lids and no pin holes were placed in the lids. The pan was heated at 10 °C/min to 120 °C and held isothermally at this temperature for 10 minutes to allow molten ketoprofen to diffuse through the polymer. The pan was then cooled at 10 °C/min to −30 °C and reheated at 5 °C/min while the temperature was modulated by 0.53 °C every 40 s. Pans were re-weighed after analysis. The glass transition onset temperature was determined from the reversing heat flow signal in the second heating cycle. This was carried out in triplicate for each system. Universal Analysis software (TA Instruments, Elstree, U.K) was used to analyze data. 

##### Prediction of Glass Transition Temperature of Polymer–Ketoprofen Systems

The predicted glass transition temperature was calculated using the Gordon–Taylor equation [21] (Equation (1)):

(1) $$T_{g}mix=\frac{\left(W_{1}T_{g1}+KW_{2}T_{g2}\right)}{\left(W_{1}+KW_{2}\right)}$$

where *T_g_mix* is the glass transition temperature of the mixed system, *T_g1_* and *W_1_* are the glass transition onset temperature and weight fraction of the first component respectively, and *T_g2_* and *W_2_* are the glass transition onset temperature and weight fraction of the second component respectively. *K* is a constant which can be calculated from Equation (2):

(2) $$K=\frac{T_{g1}\rho_{1}\;}{T_{g2}\rho_{2}}$$

where *ρ* is the true density of the material.

##### Determination of True Density by Helium Pycnometry

The density of samples was determined using an Accupyc II 1340 Pycnometer (Micromeritics, Norcross, GA, USA). Approximately 100–150 mg of the samples were accurately weighed and placed in a sample cup of 1 cm^3^ capacity where they were purged with dry helium (99.995% purity) at a pressure of 19.2 psig using an equilibration rate of 0.0050 psig/ min. Samples were analyzed in duplicate and each analysis consisted of five consecutive measurements. Results are presented as the average of ten values.

##### Determination of Relative Humidity Induced Glass Transition by DVS

The relative humidity induced glass transitions (RH_Tg_) for melt-quenched cryo-milled ketoprofen–polymer samples were determined at 25 °C (± 0.1 °C) as previously described [22]. Between 6 and 14 mg of powder were dried to a constant mass (dm/dt <0.002 mg/min) at 0% RH in the sample basket in a DVS Advantage-1 automated gravimetric vapor sorption analyzer (Surface Measurement Systems, Alperton, U.K.). After drying, the powder was exposed to a 10% RH increase per hour from 0% RH to 90% RH. From this data, a curve of percentage change in mass versus time was generated. The initial linear portion of this curve was taken to be the surface adsorption of water, and the second linear portion of this curve was taken to be a combination of surface and bulk sorption of water by the powder. The intercept of these two lines was determined and the time at which RH_Tg_ occurred was determined [22]. The relative humidity at which RH_Tg_ occurred was then determined by plotting time versus RH. A sample plot is shown in Supplementary Material, Figure S1. The amount of water sorbed by the powder at the RH_Tg_ as a percentage of starting sample mass, was also determined. Analysis was carried out in triplicate for each system.

##### Attenuated Total Reflectance Fourier-Transform Infrared Spectroscopy (ATR-FTIR)

Samples were analyzed using a Spectrum 1 FT-IR Spectrometer (Perkin Elmer, Shelton, CT, USA) equipped with a Universal Attenuated Total Reflectance and diamond/ZnSe crystal accessory. Each spectrum was scanned in the range of 650–4000 cm^−1^ with a resolution of 1 cm^−1^. The data were normalized by dividing the absorbance values in each spectrum by the maximum absorbance value recorded for that spectrum. 

#### 2.2.5. Solid State Stability 

First, 150 mg of freshly prepared samples of melt-quenched cryo-milled ketoprofen were measured into amber glass vials and placed in a vacuum sealed storage container containing either phosphorous pentoxide (0% RH) or a saturated sodium chloride solution (75% RH). These storage containers were placed in an oven (Gallenkamp, Loughborough, U.K) and maintained at 25 °C. Temperature and humidity were monitored using a logger (Sensirion, Switzerland). Samples were taken after 1, 2, 4, 8 and 12 weeks of storage for analysis by DSC and pXRD.

#### 2.2.6. Solubility Testing

##### Determination of Crystalline Ketoprofen Equilibrium Solubility at pH 1.2

An excess of ketoprofen was added to a glass amber vial with a capacity of 10 mL containing 5 mL of 0.1 M hydrochloric acid (pH 1.2). These vials were sealed and crimped and placed in a reciprocal shaking water bath (ThermoFisher Scientific, Waltham, MA, USA) maintained at 37 (±1) °C and shaken at 50 cpm. After 24 h, 1 mL of the solution was filtered using a 0.45 μm PTFE filter (Fisherbrand, Waltham, MA, USA) and diluted to an appropriate concentration in methanol before being analyzed using HPLC. All consumables which came into contact with the undiluted ketoprofen suspension were pre-heated prior to use. This was carried out for three separate vials.

##### Determination of Ketoprofen–Polymer Solid Dispersion Dynamic Solubility at pH 1.2

First, 100 mg of each polymer–ketoprofen system was added to 20 mL dilute hydrochloric acid (pH 1.2) in a jacketed beaker with a capacity of 60 mL containing maintained at 37 (±1) °C via a water bath (Lauda, Lauda-Königshofen, Germany). The suspension was stirred continuously by magnetic stirring at 1000 rpm with a 12 mm long magnet for 2 h. At various time points, 1 mL of the liquid was filtered through a 0.45 μm PTFE filter (Fisherbrand, Waltham, MA, USA), and diluted with methanol. Ketoprofen concentration was determined using HPLC. All consumables which came into contact with the undiluted ketoprofen suspension were pre-heated prior to use.

##### 2.2.7. HPLC Analysis

Ketoprofen concentration in aqueous media was determined using an Alliance HPLC with 2695 Separations Module system and 2996 photodiode array detector (Waters, Dublin, Ireland) which was used at wavelength 259 nm. The mobile phase consisted of acetonitrile and phosphate buffer pH 3 in a 45 to 55 (*v*/*v*) ratio. The phosphate buffer was prepared by adjusting the pH of a 20 mM solution of monosodium phosphate with 1 M sodium hydroxide. The column used was an ODS C18(2) column (150 mm × 4.6 mm, particle size 5 μm; Phenomenex, Le Pecq, France). Isocratic elution was used with a column temperature of 25 °C, 20 µL injection volume and a flow rate of 1 mL/min. A six-point calibration curve was created spanning 0.5 µg/mL to 500 µg/mL. Each system was tested in triplicate.

##### 2.2.8. Tabletting: Tablet Hardness, Tensile Strength and Ejection Force Measurements

Solid dispersions were mixed with MCC in a 50:50 *w*/*w* ratio in an agate pestle and mortar. Then, 200 mg of this mixture was tabletted using an NP-RD10 single punch tablet press (Natoli, St Charles, MO, USA) with an 8 mm diameter flat-faced die. The compaction pressure applied was 6 kN and the tablet was held at this pressure for 60 seconds. Tablets were removed from the die using the bottom punch and the force required to eject the tablet was recorded. Immediately after manufacture, the tablet thickness was recorded using a micrometer (AnyiMeasuring, Guilin, China) and the tablet was subjected to radial hardness testing using a handheld tablet hardness tester (Electrolab, Navi Mumbai, India). The tensile strength of the tablets was calculated using Equation (3):

σ = 2F/πDH,(3)

where σ is the tensile strength (MPa), F is the radial hardness (N), D is the tablet diameter (mm) and H is the tablet thickness (mm). 

Control measurements were also recorded for each polymer–ketoprofen system by physically mixing crystalline ketoprofen, the polymer of choice and MCC in a 10:40:50 *w*/*w* ratio in an agate pestle and mortar. For each solid dispersion and their crystalline ketoprofen controls, a minimum of 3 tablets were manufactured and tested.

## 3. Results and Discussion

### 3.1. Characterization of Raw Materials 

#### 3.1.1. Thermal Properties 

**Figure 2:**
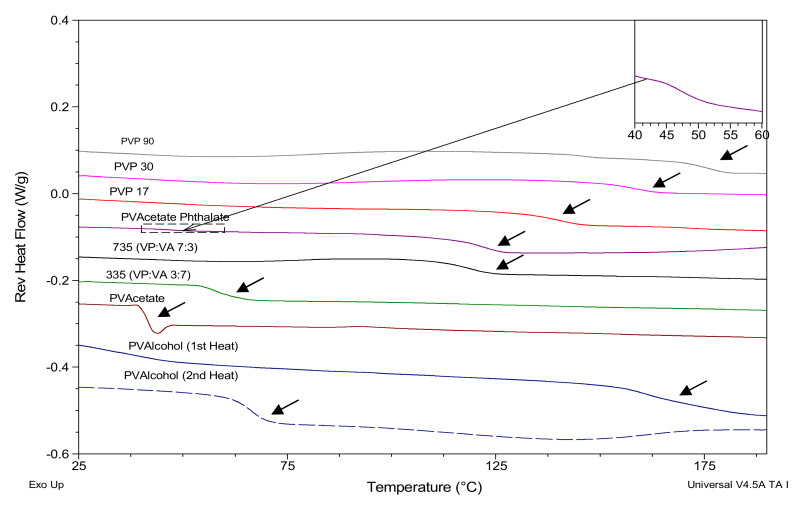
mDSC scans showing glass transitions of polymers. Inlay shows first glass transition of PVAP.

The glass transition onset temperatures of the polymers were determined using mDSC and are shown in Figure 2 and summarized in Table 2, along with the glass transition onset temperature of ketoprofen which was melt-quenched in situ in the DSC (Supplementary Material, Figure S3).

The polymer with the highest glass transition temperature is PVP90, followed by PVP30 and PVP17. As would be expected, the glass transition temperature increases with increasing molecular weight [23]. The glass transition temperature appears to reduce with increasing vinyl acetate substitution. The glass transition onset temperature of polyvinyl acetate is 38 °C, so as the substitution ratio of the vinyl acetate moiety increases, the glass transition temperature of the polymer approaches the glass transition temperature of polyvinyl acetate. 

Interestingly, polyvinyl acetate phthalate (PVAP) displays two glass transition temperatures. The first smaller glass transition temperature is at 46 °C (shown in inlay in Figure 2), while the second glass transition temperature, which has a larger heat capacity associated with it, is shown at 116 °C. As there is a vinyl acetate moiety in the PVAP structure, the lower Tg may be due to the presence of some polyvinyl acetate impurity.

PVAlcohol is the only polymer studied which is partially crystalline, and so the glass transition was determined for both the partially crystalline and the fully amorphous system, the latter being created by heating, cooling and then re-heating the polymer as shown in the Supplementary Material, Figure S2. On the first heating cycle, a small endotherm is apparent at 41 °C in the total heat flow axis. This has been attributed by other authors to the glass transition of the amorphous region of semi-crystalline PVAlcohol [24,25]. This glass transition is followed by a broad endotherm associated with the release of bound water with a peak at around 80 °C as well as a broad melting endotherm centerd at 192 °C [24]. On the second heating run, this endotherm is not apparent and the glass transition is higher, at 62 °C. This shift in Tg must be due to the incorporation of the previously crystalline portion of PVAlcohol into the amorphous region. 

A heat-cool-heat profile of ketoprofen is shown in Supplementary Material, Figure S3. Crystalline ketoprofen has a melt onset at 92 °C and no cold crystallization occurs in the cooling cycle as ketoprofen is a good glass former [26]. The glass transition onset temperature of ketoprofen is −5 °C, as seen in the second heating run. Ketoprofen remains amorphous upon reheating as evidenced by the lack of an endotherm in the second heating cycle.

#### 3.1.2. pXRD Analysis 

**Figure 3:**
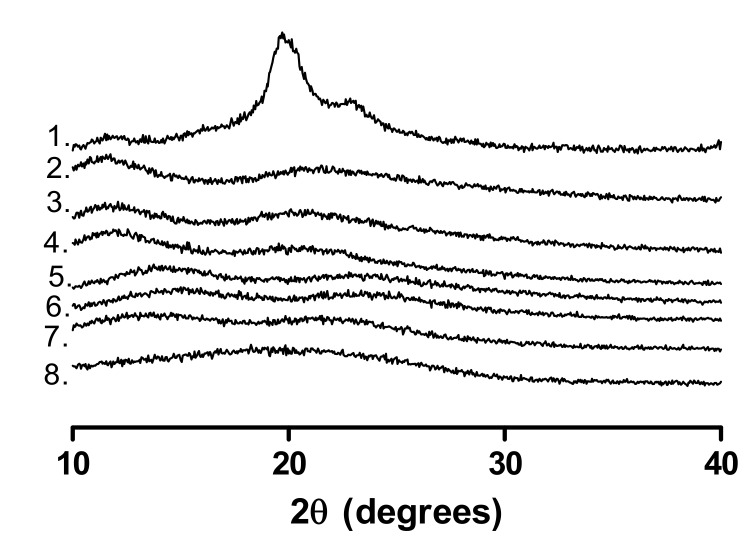
pXRD patterns of the polymers used. 1. PVAlcohol, 2. PVP90, 3. PVP30, 4. PVP17, 5. VP:VA 7:3, 6. VP:VA 3:7, 7. PVAcetate, 8. PVAP.

The pXRD patterns of the polymers are shown in Figure 3. All polymers except for PVAlcohol are amorphous, as indicated by the absence of Bragg peaks. As PVAlcohol is semi-crystalline, a Bragg peak is apparent at 20^o^ 2θ, which corresponds to the literature [27]. 

### 3.2. Polymer–Ketoprofen System Characterization 

#### 3.2.1. Thermal Properties

**Figure 4:**
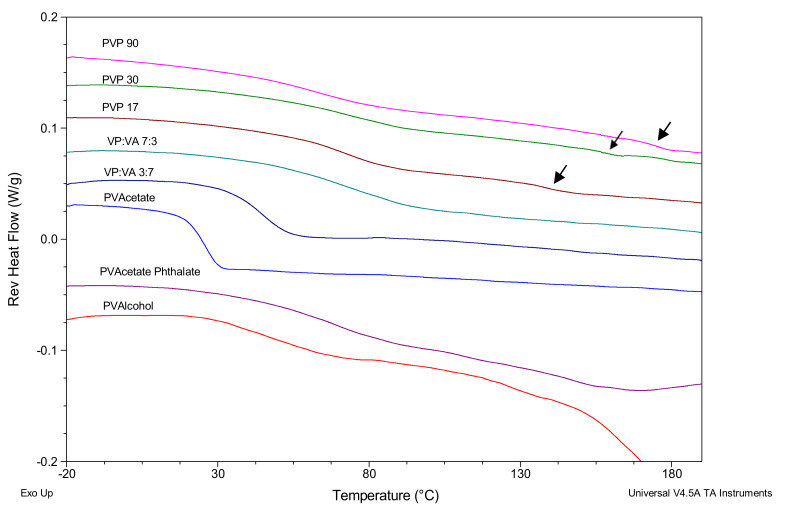
mDSC scans showing glass transitions of polymer–keto systems. Arrows point to the secondary polymer-rich glass transitions.

The DSC thermograms for polymer–keto systems with 20% *w*/*w* ketoprofen which were created by melting a physical mixture of the drug and polymer in a DSC pan are shown in Figure 4. Ketoprofen is present in the amorphous state in samples, as evidenced by the absence of a melt endotherm at 92 °C in the second heating cycle. While glass transitions are evident for all three PVP/ketoprofen (PVPKETO) systems in the 50–80 °C region, there also appears to be small secondary glass transitions present (indicated by arrows in Figure 4) in all PVPKETO systems at higher temperatures (>120 °C). This may be due to slow ketoprofen diffusion through the polymer matrix resulting in heterogenous systems, whereby there is a ketoprofen/PVP phase and a separate PVP phase. This is supported by the fact that the secondary glass transitions occur at the same temperatures as the polymer only glass transition temperatures listed in Table 2. 

By contrast, the higher vinyl-acetate containing systems have sharp, clear glass transitions with narrower glass transition widths, indicating that homogenous systems were achieved within the 10 minutes annealing time allowed for in the DSC method. This glass transition width narrowing effect appears to be more evident in systems with higher vinyl acetate content. The PVPVA/ketoprofen (PVAPKETO) system has a single, very broad glass transition, which is probably a result of the two distinct amorphous regions, as highlighted in Figure 2, mixing with amorphous ketoprofen to form one very broad glass transition. The PVAlcohol/ketoprofen (PVAlcoholKETO) system shows a glass transition onset at approximately 30 °C before the crystalline portion of PVAlcohol starts melting at 150 °C.

The predominant glass transition onset temperatures from Figure 4 were compared to values predicted by the Gordon–Taylor equation (using the true density values determined by helium pycnometry shown in Table 3). As the glass transition of ketoprofen is below room temperature, it is not possible to determine the true density of amorphous ketoprofen using traditional pycnometry. It is known that the density of amorphous compounds is lower than that of their crystalline counterparts [28]. In a previous study where pure API amorphous density measurement was not possible, the amorphous density was assumed to be 5% less than the crystalline density [29] and therefore, in this study the same assumption has been made resulting in an assumed amorphous ketoprofen density of 1.19 g/cm^3^.

**Figure 5:**
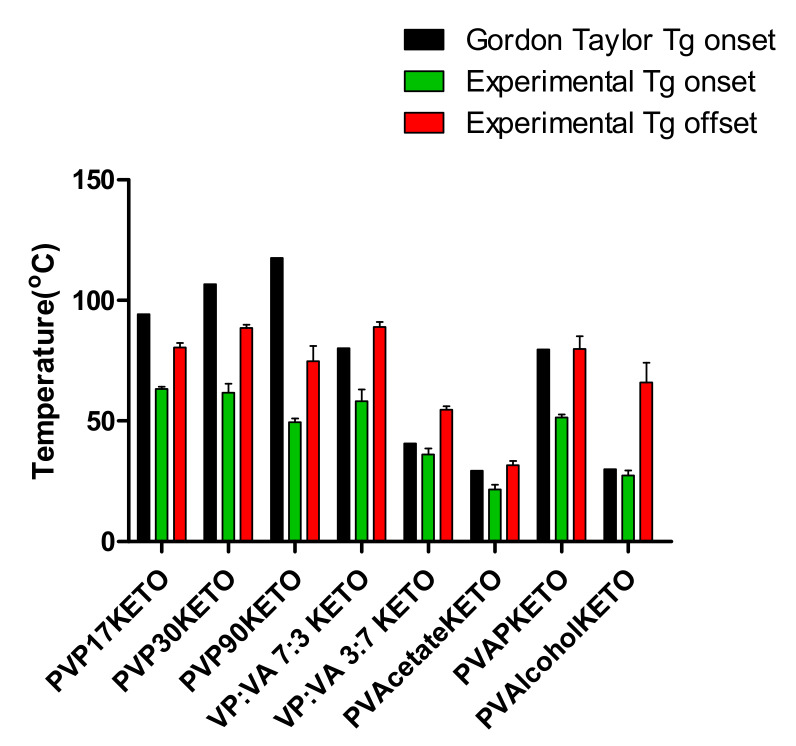
Experimental and predicted glass transition temperature values for POLYMERKETO systems with 20% *w*/*w* ketoprofen.

The deviation between experimental and predicted glass transition onset temperature of the polymer–ketoprofen systems (POLYMERKETO) systems are detailed in Supplementary Material Table S1 and graphed in Figure 5.

For many of the systems studied, there is significant deviation between the experimental and predicted Tg values. The experimental values were lower than expected for the PVP-containing systems. PVPKETO systems of high molecular weight deviated the most from predicted values in terms of an absolute deviation, up to 68 °C in the case of the PVP90KETO system. This may be partially explained by considering that the viscosity of the high molecular weight polymers may limit the diffusion of ketoprofen through the system, resulting in secondary polymer-rich glass transition temperatures, as seen in Figure 4. Systems containing vinyl acetate deviated the least from the predicted values. Initially, this difference in Gordon–Taylor deviations between vinyl acetate-rich and vinyl pyrrolidone-rich systems was thought to be due to variation of water content in the mixtures, as water is known to be a potent plasticizer [28]. However, by re-weighing the pans after the first heating cycle of the DSC measurement it was confirmed that sorbed water had evaporated despite the absence of pin-holes in the DSC lid. The mass percentage of water loss during the first heating cycle was the same as the mass percentage loss during a thermogravimetric analysis. Therefore, the systems present in the second heating cycle, where the glass transition was measured, were binary systems of drug and polymer. 

The fact that vinyl acetate-rich systems had glass transition temperature values closer to Gordon–Taylor predicted values than vinyl pyrrolidone-rich systems is somewhat surprising as the vinyl acetate functional group is thought to be a poorer hydrogen bond acceptor than vinyl pyrrolidone [30]. Generally, hydrogen bonding between the drug and polymer would be expected to produce a positive deviation from the Gordon–Taylor equation [16]. However, in specific instances where one of the components is able to self-associate (e.g. dimer formation) through hydrogen bonding while the other component can only act as a hydrogen bond donor or acceptor, the creation of an ASD involving hydrogen bond formation between the two components may lead to a net reduction in the total strength of hydrogen bonds present in the system [31]. As ketoprofen is known to self-associate to dimers through hydrogen bond formation via the carboxylic acid group [32], it may be that the negative deviation from the Gordon–Taylor equation is a result of the new hydrogen bonds formed between ketoprofen and PVP being weaker than the hydrogen bonds between amorphous dimers of ketoprofen [30]. The vinyl acetate systems experience a less negative deviation from Gordon–Taylor predicted glass transition values as they are less likely to hydrogen bond with ketoprofen and thus the strong dimer hydrogen bonds are retained. 

The PVAPKETO system’s glass transition onset temperature also negatively deviated from the value predicted by the Gordon–Taylor equation, which is likely due to its very broad glass transition (with a width of 28 °C, Supplementary Material Table S1), which, in turn, is due to the presence of a small poly vinyl acetate-rich domain, as seen in the inlay in Figure 2. As the polymer itself had two glass transitions, when the ASD was created, the addition of ketoprofen provided further heterogeneity to the system resulting in a very broad glass transition. The experimental glass transition offset temperature of PVAPKETO was very similar to the Gordon–Taylor predicted onset temperature (Figure 5). 

The PVAlcohol system glass transition temperature was very similar to the value predicted by the Gordon–Taylor equation, which is indicative of ideal mixing and the absence of any strong attractive or repulsive forces between the drug and polymer. 

One limitation with Gordon–Taylor predicted glass transition values is that they are generally understood to refer to the glass transition onset temperature. Where glass transition temperatures occur over an extended range, such as is the case with many of the polymer–ketoprofen systems, the Gordon–Taylor predicted glass transition temperature may fall within the experimental glass transition range but deviate significantly from the glass transition onset temperature.

#### 3.2.2. pXRD Analysis

**Figure 6:**
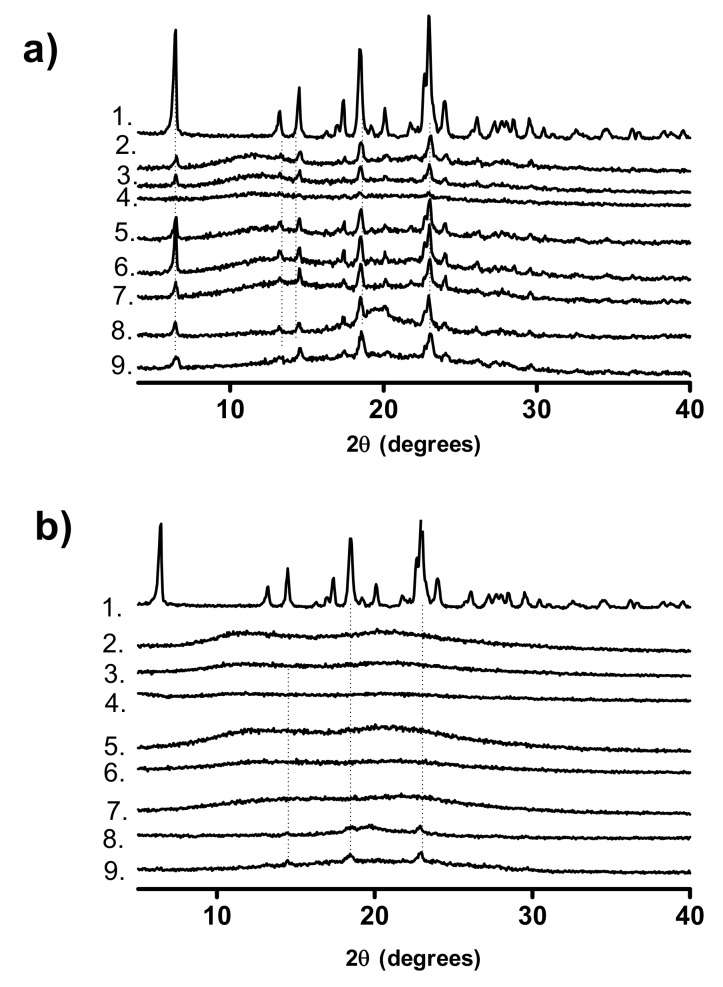
pXRD patterns of (**a**) Physical mixtures of crystalline ketoprofen and polymers, (**b**) Melt-quenched and cryo-milled POLYMERKETO. 1. Crystalline ketoprofen, 2. PVP90KETO, 3. PVP30KETO, 4. PVP17KETO, 5. VP:VA 7:3KETO, 6. VP:VA 3:7KETO, 7. PVAcetateKETO, 8. PVAlcoholKETO, 9. PVAPKETO.

The pXRD patterns of physical mixtures of crystalline ketoprofen and the studied polymers are shown in Figure 6a. The pXRD pattern of the PVP with the lowest molecular weight, PVP17, physically mixed with ketoprofen appears to have Bragg peaks which are of lower intensity relative to the equivalent higher molecular weight PVP physical mixtures. This observation has been reported by other authors for ibuprofen [33]. One explanation for this observation is that the lower molecular weight PVPs have a higher number of terminal groups which are less sterically hindered and are therefore freer to interact with the carboxylic acid group of ketoprofen, resulting in a disturbance of the crystal packing [33]. It should also be considered that the low drug loading in these systems (20% *w*/*w*) means that even small variations in sample size may reduce the relative intensity of the Bragg peaks. 

The pXRD patterns shown in Figure 6b are the same systems post melt-quenching and cryo-milling. While all physical mixtures show Bragg peaks corresponding to crystalline ketoprofen, once melt-quenched all systems, except PVAPKETO and PVAlcoholKETO, are pXRD amorphous with no Bragg peaks visible. 

For both PVAPKETO and PVAlcoholKETO, the intensity of the Bragg peaks is reduced in the melt-quenched systems relative to the physical mixture, indicating some degree of amorphization, but also that residual crystalline ketoprofen remains. This is interesting as DSC showed no crystalline ketoprofen endotherm for the in situ melt-quenched systems (Section 3.2.1). This may be explained by consideration that the DSC pan, where the glass transition measurements were taken, involves the use of a much smaller sample size in a very controlled environment in comparison to the melt-quenched system, which was created using a hot-plate and liquid nitrogen. 

Alternatively, the presence of residual crystalline ketoprofen in both systems may be due to rapid re-crystallization of ketoprofen in the time between amorphization via melt-quenching and recording of the pXRD pattern. Another explanation may be that ketoprofen may be soluble at this drug/polymer ratio (20% *w*/*w* ketoprofen) for both polymers at the elevated temperatures used in the DSC method and hence no crystalline endotherm is visible. The ketoprofen solubility limit may be lower for these polymers at the ambient temperatures used for pXRD.

#### 3.2.3. ATR-FTIR Analysis 

The intermolecular bonding between poly-vinyl-based polymers and ketoprofen was investigated using ATR-FTIR. Intermolecular interactions or lack thereof are crucial in the understanding of the polymer mediated stabilization of amorphous APIs. It is known that the vinyl pyrrolidine functional group in PVP can hydrogen bond with the carboxylic acid carbonyl in ketoprofen, and that, in the PVPVA 6:4 co-polymer, the vinyl pyrrolidine functional group rather than the vinyl acetate group is preferentially involved in hydrogen bonding with the carboxylic acid carbonyl group of ketoprofen [34]. In the current study, we aimed to investigate if vinyl acetate, vinyl alcohol or vinyl phthalate functional groups can interact with the carbonyls of the carboxylic acid or the ketone of ketoprofen. 

**Figure 7:**
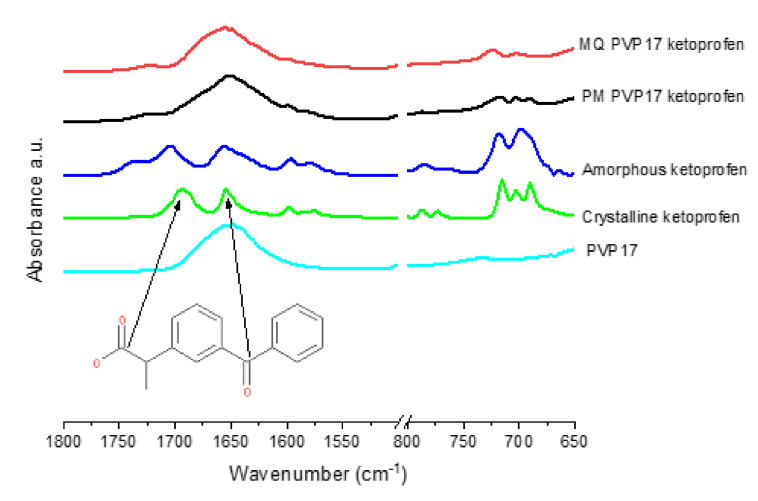
PVP17 and ketoprofen ATR-FTIR spectra. Melt-quenched (MQ) PVP17KETO and physical mixture (PM) PVP17KETO.

In crystalline ketoprofen (Figure 7), there are two carbonyl signals at 1694 and 1653 cm^−1^, corresponding to the dimer hydrogen bonded carbonyl group of the carboxylic acid and the carbonyl of the ketone respectively [34,35]. In amorphous ketoprofen, the dimer carbonyl is slightly shifted to a higher wavenumber (blue shift) at 1705 cm^−1^, indicating that intermolecular hydrogen bonding is stronger in the crystalline state of ketoprofen than the amorphous state, as hydrogen bonding results in a lengthening of the carbonyl bond and lowers the frequency and hence wavenumber of the signal [36,37]. The ketone carbonyl at 1655 cm^−1^ in the amorphous ketoprofen sample is broader than that in the crystalline material, indicating amorphization [36]. Additionally, notable in the amorphous spectrum is the appearance of a shoulder at 1737 cm^−1^, which may be assigned to the free acid (i.e., COOH) carbonyl of the carboxylic acid group, and is visible due to the presence of monomeric ketoprofen [34,35,36]. Another observable difference between the crystalline and amorphous ketoprofen is noted in the fingerprint region. In crystalline ketoprofen, there are 3 distinct peaks between 720 and 650 cm^−1^, while in amorphous ketoprofen, there are only 2 peaks visible in this region, which allows for this region to be used as an indicator of the solid state nature of ketoprofen [34]. 

As shown in Figure 7 and Supplementary Material Figures S4 and S5, PVP has one strong signal in the carbonyl region centered at 1653 cm^−1^, corresponding to the carbonyl of the vinyl pyrrolidone ring. The physical mixtures (PM) of PVP17, PVP30 and PVP90 with 20% *w*/*w* ketoprofen appear to simply be an overlay of the IR spectra of each component, with the 3 peaks attributed to crystalline ketoprofen visible in the fingerprint region.

In contrast, the melt-quenched (MQ) samples show 2 peaks in the fingerprint region, indicating amorphization of ketoprofen. The melt-quenched samples also show a red shift of the dimer carboxylic acid peak of ketoprofen, causing it to overlap with the ketone of ketoprofen and the vinyl pyrrolidone peak, which indicates that amorphization is associated with an increase in hydrogen bonding between ketoprofen and PVP. The amorphous ketoprofen monomeric shoulder is also present but is slightly shifted to lower wavenumbers at 1725, 1723 and 1726 cm^−1^ for PVP17KETO PVP30KETO and PVP90KETO, respectively which is indicative of increased hydrogen bonding relative to ketoprofen in the amorphous state. It is clear therefore that melt-quenching PVP with 20% *w*/*w* ketoprofen results in an amorphous material which is characterized by increased hydrogen bonding relative to the physical mixture. These spectra are complementary to the mDSC results which showed that PVPKETO systems exhibited glass transition onset temperatures which deviated significantly from the Gordon–Taylor equation. It is clear from ATR-FTIR that hydrogen bonding is occurring between the ketoprofen carboxylic carbonyl and PVP and the ability of PVP to form hydrogen bonds with ketoprofen at the expense of stronger ketoprofen–ketoprofen hydrogen bonds explains the negative deviation of glass transition temperature from predicted values.

As shown in Supplementary Material Figure S6, VP:VA 7:3 has two carbonyl peaks apparent, a peak of low intensity at 1732 cm^−1^ corresponding to vinyl acetate and a peak of higher intensity at 1676 cm^−1^ corresponding to vinyl pyrrolidone. The physical mixture of polymer VP:VA 7:3 and 20% *w*/*w* ketoprofen shows two distinct peaks, one at 1735 cm^−1^ corresponding to the vinyl acetate group and one centered at 1671 cm^−1^ corresponding to the merging of the ketone of ketoprofen, dimer carbonyl of ketoprofen and vinyl pyrrolidone. In the melt-quenched sample, the fingerprint region shows two distinct peaks in the 650–720 cm^−1^ region, indicating amorphization, however there is no clear change in the carbonyl region. The vinyl acetate signal remains unchanged, indicating that it is not involved in hydrogen bonding with ketoprofen. 

In a similar manner, ATR-FTIR spectra of the VP:VA 3:7 co-polymer melt-quenched system (Supplementary Material Figure S7), and the PVAcetate (Supplementary Material Figure S8), PVAlcohol (Supplementary Material Figure S9) and PVAP (Supplementary Material Figure S10) melt-quenched systems indicate ketoprofen amorphization (based on the fingerprint region) but no change in the position of the vinyl acetate or alcohol signals, relative to the physical mixtures. 

Thus, the ATR-FTIR analysis has shown that ketoprofen is ATR-FTIR amorphous for all of the melt-quenched systems studied. No shifts were observed with any vinyl acetate or vinyl alcohol groups indicating that these functional groups do not form hydrogen bonds with ketoprofen in the amorphous form. The ASDs with high VA content demonstrated glass transition temperatures which were closest to the Gordon–Taylor predicted values (Figure 5), which is due to their low propensity to form hydrogen bonds with ketoprofen, allowing the stronger dimeric ketoprofen hydrogen bonds to remain intact. The only systems in which there was clear evidence of hydrogen bonding between ketoprofen and polymer were the PVPKETO systems, which complements DSC findings. 

### 3.3. Influence of Polymer Choice on Water-Induced Phase Transition

**Figure 8:**
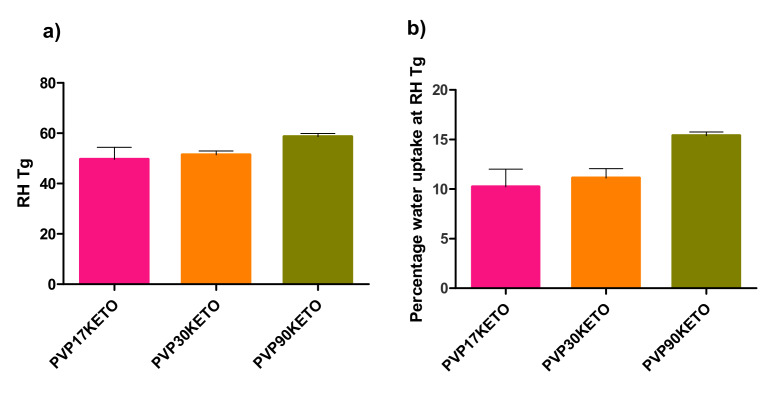
(**a**) The relative humidity at which a glass transition was induced for PVP–ketoprofen systems of differing molecular weights. (**b**) The mass percentage of water uptake for PVP–ketoprofen systems at the relative humidity induced glass transition for differing PVP molecular weights.

**Figure 9:**
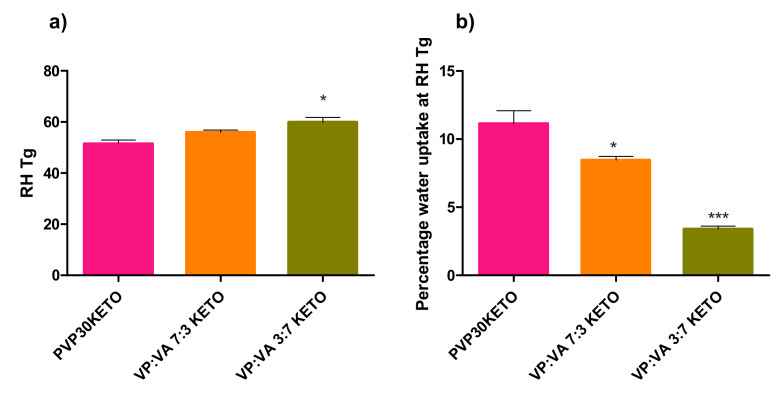
(**a**) The relative humidity at which a glass transition was induced for polymer–ketoprofen systems of differing VP:VA substitution ratios. (**b**) The mass percentage of water uptake for polymer–ketoprofen systems at the relative humidity induced glass transition for differing VP:VA substitution ratios. Statistically significant difference (*p* < 0.05) from PVP30KETO system denoted by *. Highly statistically significant difference (*p* < 0.001) from PVP30KETO system denoted by ***.

The relative humidity at which the relative humidity induced glass transition (RH_Tg_) occurred, as well as the percentage of water uptake at the RH_Tg_, were determined as described in Section 2.2.4 and are shown in Figure 8 and Figure 9. The RH_Tg_ is the relative humidity at which sufficient water has been adsorbed by the system for the glass transition to occur at the temperature of the analysis [22]. 

As PVAPKETO and PVAlcoholKETO are partially crystalline (by pXRD), they were not deemed suitable for this analysis. Similarly, as this analysis was carried out at 25 °C, which is above the glass transition temperature of PVAcetateKETO, this system was not included in this analysis. 

RH_Tg_ is useful as it gives an indicator as to how susceptible an ASD system may be to moisture-induced phase changes. As moisture is sorbed, the glass transition of the system reduces due to the plasticization by water. If enough moisture is sorbed, the system transitions from the amorphous to the supercooled liquid state. The purpose of this section of work was to examine whether or not the physicochemical properties of the polymer influenced the RH at which RHTg was reached for the ASD systems studied.

#### 3.3.1. Influence of Polymer Molecular Weight on Water-Induced Phase Transition 

There was no statistically significant difference in the RH_Tg_ between the PVP30KETO system and the other molecular weight PVP systems studied. A trend is apparent however, as shown in Figure 8a. As the molecular weight of PVP increases, the RH required for RH_Tg_ to occur increases. The mean RH_Tg_ (± standard error) for PVP17KETO, PVP30KETO and PVP90KETO was 49.61 ± 4.83, 51.44 ± 1.54 and 58.71 ± 1.16 %RH, respectively. This is likely due to the effect that increasing molecular weight has on increasing the thermal glass transition (Tg = 136 °C, 154 °C and 172 °C for PVP17, 30 and 90 respectively, Table 2). The higher molecular weight PVP systems therefore require a higher RH to be reached before the amount of water sorbed plasticizes the system to a sufficient degree to enable a moisture induced glass transition to occur at 25 °C. The higher molecular weight may mean that the water has more difficulty diffusing through the powder due to reduced molecular mobility caused by the high viscosity of the polymer.

#### 3.3.2. Influence of Polymer Substitution Ratio on Water-Induced Phase Transition 

Similarly, a trend is apparent in the RH_Tg_s of systems with different VP:VA ratios studied, as shown in Figure 9. As the ratio of VP:VA increases, the RH_Tg_ reduces. The RH_Tg_ values (± standard error) for VP:VA ratios of 10:0, 7:3 and 3:7 are 51.44 ± 1.54, 55.98 ± 0.88 and 59.93 ± 1.84 %RH respectively. Increasing the vinyl acetate proportion in the system increases the hydrophobicity of the system and therefore higher RH is required for sufficient water to be adsorbed for phase transition to occur. In contrast to Figure 8 where higher RH_Tg_ systems had higher percentages of water uptake at RH_Tg_, in Figure 9, higher RH_Tg_ systems had lower percentages of water uptake at RH_Tg_. This is explained by consideration of the thermal glass transitions of these systems. The glass transition of polyvinyl acetate is 38 °C, and as the proportion of vinyl acetate increases the glass transition of the co-polymer reduces. The glass transitions of PVP30 (VP:VA 10:0), VP:VA 7:3 and VP:VA 3:7 are 154, 114 and 58 °C respectively. In the case of the VP:VA 7:3 KETO system, only 3.39% *w*/*w* water was required for RH_Tg_ to occur, but its hydrophobicity necessitated the RH to be nearly 60% before this amount of water could be sorbed. Where no vinyl acetate was present, 11.13% *w*/*w* water was required for RH_Tg_ to occur, but the hydrophilicity of the system meant that this quantity of water was sorbed by the time the system reached 51% RH.

As the data above refer to the RH_Tg_ at one relative humidity ramping rate (10% RH/ hour), it cannot be used to determine the critical relative humidity for these polymer–keto systems as this requires experiments at several relative humidity ramping rates to be performed. The critical relative humidity of a system is the humidity above which a glass transition will occur at a particular temperature given sufficient time to sorb moisture [22]. This can be used to prescribe storage conditions for the ASD. However, it is clear from the data outlined above that higher polymer molecular weight and hence higher viscosity is protective against moisture induced phase transition, as is increasing the hydrophobic vinyl acetate component in the co-polymer.

### 3.4. Influence of Polymer Choice on Dynamic Solubility

**Figure 10:**
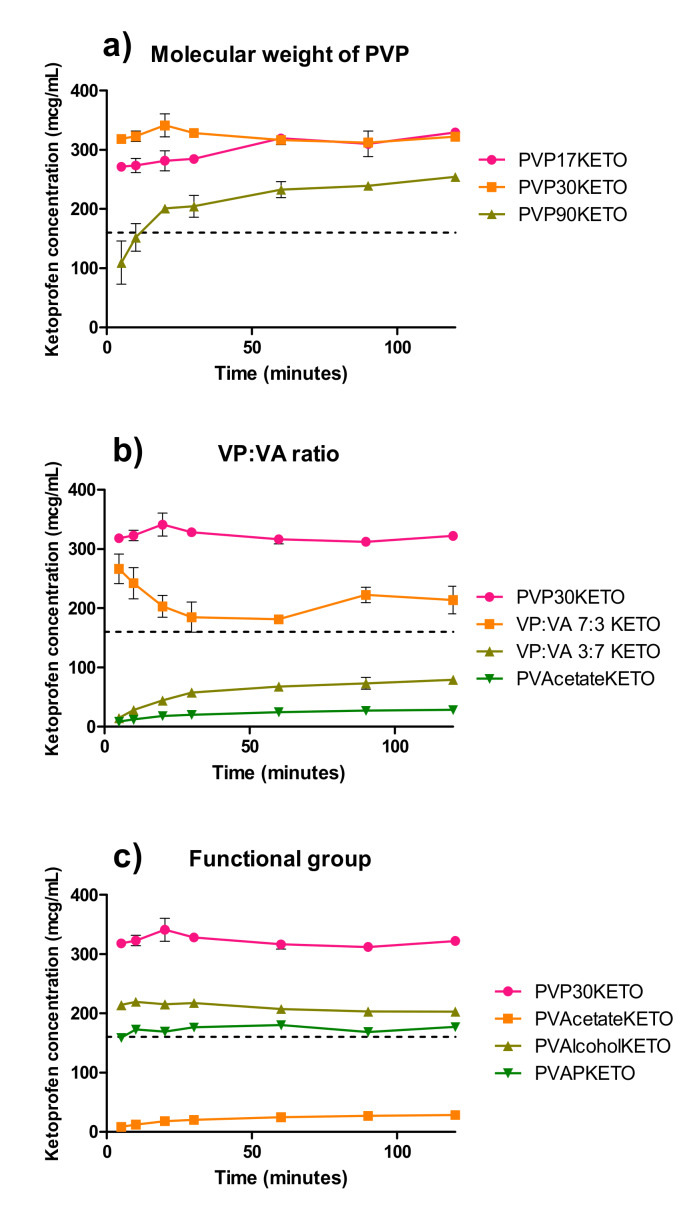
Dynamic solubility of POLYMERKETO systems at pH 1.2 and 37 °C over two hours for (**a**) PVPKETO systems, (**b**) VP:VA KETO systems and (**c**) PVAlcoholKETO and PVAPKETO systems. The dashed line represents the equilibrium solubility of crystalline ketoprofen.

The equilibrium solubility of ketoprofen in pH 1.20 at 37 °C was determined to be 0.157 ± 0.029 mg/mL at 24 h. This value is close to another the value reported in the literature of 0.130 mg/mL [38]. The dynamic solubility of the polymer–ketoprofen systems are shown in Figure 10, where the dashed line represents the equilibrium solubility of crystalline ketoprofen.

As seen in Figure 10a all PVP–ketoprofen systems reach supersaturation relative to crystalline ketoprofen within two hours. The PVP90KETO system was slower to reach supersaturation than the lower molecular weight PVPKETO systems, which is likely due to the high viscosity of this polymer. The total degree of supersaturation is lower for PVP90KETO relative to the other two PVP polymers over two hours, although it is clear the degree of supersaturation is increasing with time. If the experiment had been conducted over a longer time frame, PVP90KETO may have attained the same degree of supersaturation as the lower molecular weight PVP systems, but this would not have been physiologically relevant. Interestingly, PVP30KETO reached supersaturation faster than PVP17KETO, which is a surprising result considering that the viscosity of PVP17 is lower than that of PVP30. 

The ratio of vinyl pyrrolidone to vinyl acetate is clearly critical to the ability of ketoprofen ASDs to reach supersaturation, as shown in Figure 10b. When this ratio reached 3:7 VP:VA or lower, the ASD was no longer able to reach supersaturation within two hours. This is due to the inherently low aqueous solubility of the vinyl acetate functional group [15]. As shown in Supplementary Material Figure S11, the relationship between vinyl pyrrolidone composition and ketoprofen concentration after two hours is well described by a linear regression model, with an R^2^ value of 0.96. 

A similar study, which examined the relationship between celecoxib supersaturation and PVPVA substitution ratio for celecoxib ASDs, found that while the vinyl pyrrolidone monomer was responsible for supersaturation, the vinyl acetate monomer was responsible for the prevention of recrystallization during dissolution [39]. In the absence of API recrystallization in the dissolution media, the degree of supersaturation appears to be dependent on the degree of vinyl pyrrolidone substitution. This dependency on vinyl pyrrolidone content for ketoprofen dissolution is an interesting observation which may be explained by the concept of congruent dissolution of drug and polymer, which is known to be aided by the presence of intermolecular interactions [40,41]. This argument is strengthened by the evidence of hydrogen bonding between PVP and ketoprofen which has been provided by the FTIR data. 

The role that the polymer’s substituent functional groups have on the degree of supersaturation achieved by ketoprofen is demonstrated in Figure 10c. While PVAlcoholKETO and PVAPKETO contained ketoprofen in a mixture of amorphous and crystalline states, these systems achieved supersaturation relative to the fully crystalline ketoprofen. This is in contrast to the PVAcetateKETO system, which, although fully amorphous, did not achieve supersaturation of ketoprofen within 2 hours at pH 1.20. This work has highlighted that it is the aqueous solubility of the polymer, rather than the degree of amorphization, which is critical to the ability of ketoprofen ASDs to achieve supersaturation. 

### 3.5. Influence of Polymer Choice on the Stability of the Glassy State

The pXRD patterns of POLYMERKETO systems placed in stability chambers maintained at 25 °C and a constant humidity of 0% RH or 75% RH over a period of 12 weeks are shown in Supplementary Material Figure S12. pXRD patterns of a physical mixture of ketoprofen (20% *w*/*w*) and the polymer being tested are also shown for comparison (purple diffractogram in each graph). All PVPKETO systems remained amorphous regardless of polymer molecular weight or the humidity at which the system was stored. Similarly, the VP:VA KETO and PVAcetateKETO systems remained amorphous regardless of storage conditions. The PVAlcoholKETO system, which had some Bragg peaks corresponding to crystalline ketoprofen at the time of manufacture, remained in a semi-crystalline state throughout the 12-week study without any significant change in peak intensity for samples stored at 0% RH and 75% RH. The PVAPKETO system, which had some small Bragg peaks corresponding to crystalline ketoprofen at the time of manufacture, displays peaks which are similar in intensity at 12 weeks in the sample stored at 0% RH and peaks which are slightly more intense at 12 weeks in the sample stored at 75% RH. 

**Figure 11:**
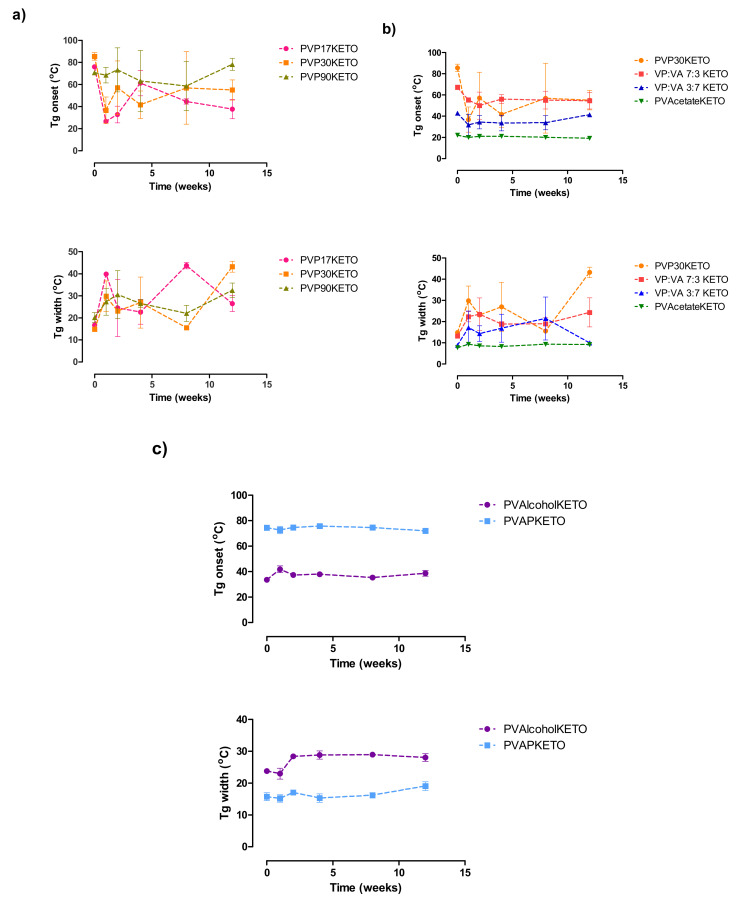
Glass transition onset temperatures and glass transition temperature width of (**a**) PVPKETO, (**b**) VP:VA KETO and (**c**) PVAlcoholKETO and PVAPKETO systems stored at 75% RH at 25 °C for 12 weeks.

The glass transition onset temperatures and glass transition temperature widths of the same systems stored at 75% RH are shown in Figure 11. The PVPKETO system which had the most significant glass transition onset temperature shift upon exposure to humidity was the PVP17KETO system (Figure 11a). While the glass transition onset temperature of PVP17KETO, PVP30KETO and PVP90KETO were all similar after manufacture, after 12 weeks exposure to 75% RH, the lower molecular weight PVP has a glass transition onset temperature which is more depressed, indicating increased water sorption relative to the other systems. Interestingly, the glass transition temperature widths, which may be considered a measure of the homogeneity of the system [42], increased for all PVPKETO systems after 12 weeks exposure to moisture. This is likely due to the presence of moisture causing the system to become a ternary system of water, polymer and ketoprofen, causing heterogeneity in the sample. 

Figure 11b shows the glass transition onset temperatures and glass transition temperature ranges of the VP:VA KETO systems as well as the PVAcetateKETO and PVP30KETO systems. Directly after manufacturing, the systems with higher vinyl pyrrolidone content have higher glass transition onset temperatures. After 12 weeks of storage at 75% RH, the glass transition onset temperatures of the high vinyl actetate content samples (i.e. VP:VA 3:7 KETO and PVAcetateKETO) have not changed, while those with high vinyl pyrrolidone content (VP: VA 7:3 KETO AND PVP30KETO) have decreased. This is due to water sorption of the systems which depresses the glass transition temperature. 

Interestingly, the glass transition onset temperatures of the PVP30KETO system and the VP:VA 7:3 KETO system were very similar after 12 weeks. The glass transition temperature width of the high vinyl pyrrolidone content systems also increased substantially over the 12-week time frame. The PVAlcohol and PVAP systems had no change in glass transition temperature over the time frame studied and a much smaller increase in glass transition temperature width relative to the other systems studied, as shown in Figure 11c.

### 3.6. Influence of Polymer Choice on the Processability of ASDs—Tensile Strength and Ejection Force

**Figure 12:**
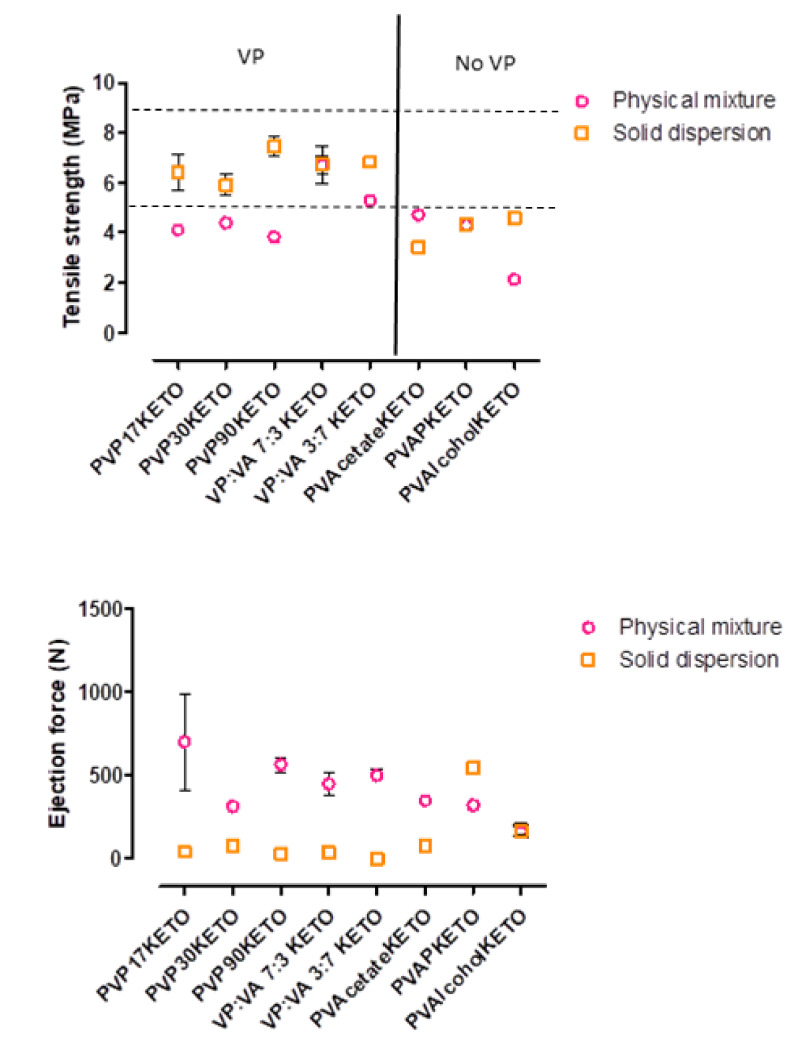
Tensile strength and ejection force of tablets formed from POLYMERKETO solid dispersion systems and equivalent physical mixtures.

The tensile strength of tablets formed from the melt-quenched cryo-milled powders combined with MCC in a 1:1 *w*/*w* ratio, as well as the ejection force required to remove the tablet from the die are shown in Figure 12. A physical mixture of polymer: ketoprofen: MCC 4:1:5 *w*/*w*/*w* was also tableted for reference.

In general, the solid dispersion tablets have higher tensile strength and require less force for die-ejection than the physical mix, with some notable exceptions. The VP:VA 7:3 KETO and PVAPKETO solid dispersion tablets had very similar tensile strengths to their equivalent physical mixtures. The PVAcetateKETO solid dispersion tablets were weaker than their equivalent physical mixture. This may be explained by considering that the PVAcetateKETO system has a glass transition onset temperature (21 °C) very close to room temperature and therefore exists in the rubbery state at test conditions. This may mean that the mechanism by which the tablet deforms and breaks is different from the equivalent crystalline or amorphous state. This was observed experimentally as the PVAcetateKETO tablets were malleable and the force applied by the hardness testing machine resulted in tablet deformation rather than breakage, in contrast to what was observed for all other systems. In addition to this, it was observed that the PVAcetateKETO system formed large rubbery clumps when allowed to equilibrate to room temperature, and as tensile strength is known to be inversely related to particle size [43], this result is not entirely unexpected. 

The relationship between tensile strength and particle size may also explain how most of the POLYMERKETO solid dispersion system tablets were stronger than their equivalent physical mixture as they were milled as part of the method used to produce them. Another explanation for the solid dispersion tablet strength superiority may be that amorphous particles have been shown to have higher effective interparticulate contact area and stronger interparticulate bonding than crystalline particles, as has been shown to be the case for lactose [44]. This results in stronger compacts. 

No trend was observed with regard to polymer molecular weight or substitution ratio and tablet tensile strength. However, the POLYMERKETO systems containing the vinyl pyrrolidone group (PVP17KETO, PVP30KETO, PVP90KETO, VP:VA 7:3 KETO, VP:VA 3:7 KETO) had higher tensile strengths than the systems which did not have this functional group. As PVP is also used as a binder excipient in formulations, and increasing the binder concentration is known to increase tablet hardness [45], the vinyl pyrrolidone moiety in solid dispersions has a dual functionality as a polymeric carrier but also as a binder.

## 4. Conclusions

The advantages and disadvantages associated with different poly-vinyl polymer properties for formulating ASDs of ketoprofen, as demonstrated in this work, are summarized in Table 4 below. There are many factors which must be considered when selecting a polymer for an ASD dispersion. While the PVP polymers have proven to be very popular in ASD development, their desirable high glass transition temperature may be offset during storage due to their propensity to sorb moisture. Identifying the optimal VP:VA ratio will help to ensure that achieving excellent aqueous solubility does not jeopardize the solid-state stability of the amorphous state. It is clear from the studies presented here that the optimal VP:VA ratio for ketoprofen ASDs lies somewhere between 3:7 and 7:3. While the PVPVA polymer which is commonly used in ASD systems has a VP:VA ratio (6:4) which falls in between these ratios, it would be interesting to examine the effect that 4:6 and 5:5 VP:VA systems have on ASD properties to identify if the commercially used polymer could be improved upon.

## Figures and Tables

**Table 1:** Molecular weights and sources of polymers.

Polymer	Supplier
Polyvinyl acetate **(PVAcetate)** Mw 100,000 g/mol	Acros Organics (Geel, Belgium)
Polyvinyl acetate phthalate **(PVAP)** Mw 47,000–61,000 g/mol [15]	Colorcon (Indianapolis, IN, USA)
Polyvinyl alcohol **(PVAlcohol)** Mw 15,000 g/mol	MP Biomedicals (Irvine, CA, USA)
Polyvinyl pyrrolidone K17 **(PVP 17)** Mw 7000–11,000 g/mol	BASF (Ludwigshafen, Germany)
Polyvinyl pyrrolidone K30 **(PVP 30)** Mw 54,000–55,000 g/mol	BASF (Ludwigshafen, Germany)
Polyvinyl pyrrolidone K90 **(PVP 90)** Mw 1,000,000–1,500,000 g/mol	BASF (Ludwigshafen, Germany)
Polyvinyl pyrrolidone vinyl acetate 3:7 E335 **(VP:VA 3:7)** Mw 28,000 g/mol [20]	Ashland (Lexington, KY, USA)
Polyvinyl pyrrolidone vinyl acetate 7:3 E735 **(VP:VA 7:3)** Mw 56,700 g/mol [20]	Ashland (Lexington, KY, USA)

**Table 2:** Thermal event values (± standard deviation) of raw materials.

Polymer	Tg	Tm (onset)
PVP 90	172.0 ± 1.2 °C	
PVP 30	153.6 ± 2.1 °C	
PVP 17	135.6 ± 0.3 °C	
PVPVA 7:3	114.2 ± 1.1 °C	
PVPVA 3:7	57.9 ± 4.3 °C	
PVAcetate	38.6 ± 1.4 °C	
PVAP	46.3 ± 0.7 °C / 116.0 ± 0.6 °C	
PVAlcohol 1st heating run	41.1 ± 3.9 °C	155.0 ± 0.1 °C
PVAlcohol 2nd heating run	64.51 ± 15 °C	

**Table 3:** True density values of ketoprofen and studied polymers. Values are presented as an average ± standard deviation. * Value determined by reducing the crystalline density by 5%.

Material	True Density (g/cm^3^)
Crystalline ketoprofen	1.25 ± 0.01
Amorphous ketoprofen^*^	1.19 ± 0.01
PVP 17	1.20 ± 0.00
PVP 30	1.20 ± 0.00
PVP 90	1.24 ± 0.00
VP:VA 7:3	1.21 ± 0.00
VP:VA 3:7	1.23 ± 0.00
PVAcetate	1.22 ± 0.00
PVAlcohol	1.31 ± 0.00
PVAP	1.38 ± 0.02

**Table 4:** Summary of the effects of poly-vinyl polymer properties on polymer–ketoprofen ASD performance.

Poly-Vinyl Polymer Property	Effect on ASD Performance
High Mw	Higher glass transition temperature More resistant to water induced phase transition Slower to reach supersaturation
High VP content	Higher glass transition temperature More prone to water induced phase transition Higher degree of supersaturation Higher propensity to hydrogen bond with the API Any VP content associated with higher tablet tensile strength
High VA content	Lower glass transition temperature Less prone to water induced phase transition Lower degree of supersaturationLower propensity to hydrogen bond with the API

## Supplementary Materials

The following are available online at https://www.mdpi.com/1999-4923/12/5/433/s1, Figure S1: Sample plot of RHTg determination for melt-quenched cryo-milled PVP30KETO system, Figure S2: Differential scanning calorimetry scans showing glass transitions/melt endotherm of PVAlcohol, Figure S3: mDSC calorimetry scans showing melt endotherm and glass transition of ketoprofen, Figure S4: PVP30 and ketoprofen ATR-FTIR spectra. Melt-quenched (MQ) PVP30KETO and physical mixture (PM) PVP30KETO, Figure S5: PVP90 and ketoprofen ATR-FTIR spectra. Melt-quenched (MQ) PVP90KETO and physical mixture (PM) PVP90KETO, Figure S6: PVP:VA 7:3 and ketoprofen ATR-FTIR spectra. Melt-quenched (MQ) VP:VA 7:3 KETO and physical mixture (PM) VP:VA 7:3 KETO, Figure S7: PVP:VA 3:7 and ketoprofen ATR-FTIR spectra. Melt-quenched (MQ) VP:VA 3:7 KETO and physical mixture (PM) VP:VA 3:7 KETO, Figure S8: PVAcetate and ketoprofen ATR-FTIR spectra. Melt-quenched (MQ) PVAcetate KETO and physical mixture (PM) PVAcetate KETO, Figure S9: PVAlcohol and ketoprofen ATR-FTIR spectra. Melt-quenched (MQ) PVAlcohol KETO and physical mixture (PM) PVAlcohol KETO, Figure S10: PVAP and ketoprofen ATR-FTIR spectra. Melt-quenched (MQ) PVAP KETO and physical mixture (PM) PVAP KETO, Figure S11: Dynamic solubility of vinyl pyrrolidone KETO systems at pH 1.2 at 2 hours versus parts vinyl pyrrolidone in the polymer, Figure S12: a) pXRD patterns of PVPKETO systems stored at 0% or 75% RH at 25 °C for 12 weeks b) pXRD patterns of VP: VA 7:3 KETO, VP: VA 3:7 KETO and PVAcetateKETO systems stored at 0% or 75% RH at 25 °C for 12 weeks c) pXRD patterns of PVAPKETO and PVAlcoholKETO systems stored at 0% or 75% RH at 25 °C for 12 weeks, Table S1: Gordon–Taylor predicted glass transition onset temperatures and experimental glass transition onset and offset temperatures and glass transition temperature widths for POLYMERKETO systems with 20% *w*/*w* ketoprofen. Values are presented as an average ± standard deviation.
