# Supplementary material for: Physicochemical Properties of Poly-vinyl Polymers and Their Influence on Ketoprofen Amorphous Solid Dispersion Performance: A Polymer Selection Case Study
Source: Pharmaceutics. 2020 May 8;12(5):433. doi: 10.3390/pharmaceutics12050433 (PMC7284699; doi:10.3390/pharmaceutics12050433)
Supplement: Supplementary file 1 [file pharmaceutics-12-00433-s001.pdf]

pharmaceutics

Supplementary Materials: Physicochemical Properties of Poly-vinyl Polymers and Their Influence on Ketoprofen Amorphous Solid Dispersion Performance: A Polymer Selection Case Study

Emer Browne, Zelalem A. Worku and Anne Marie Healy

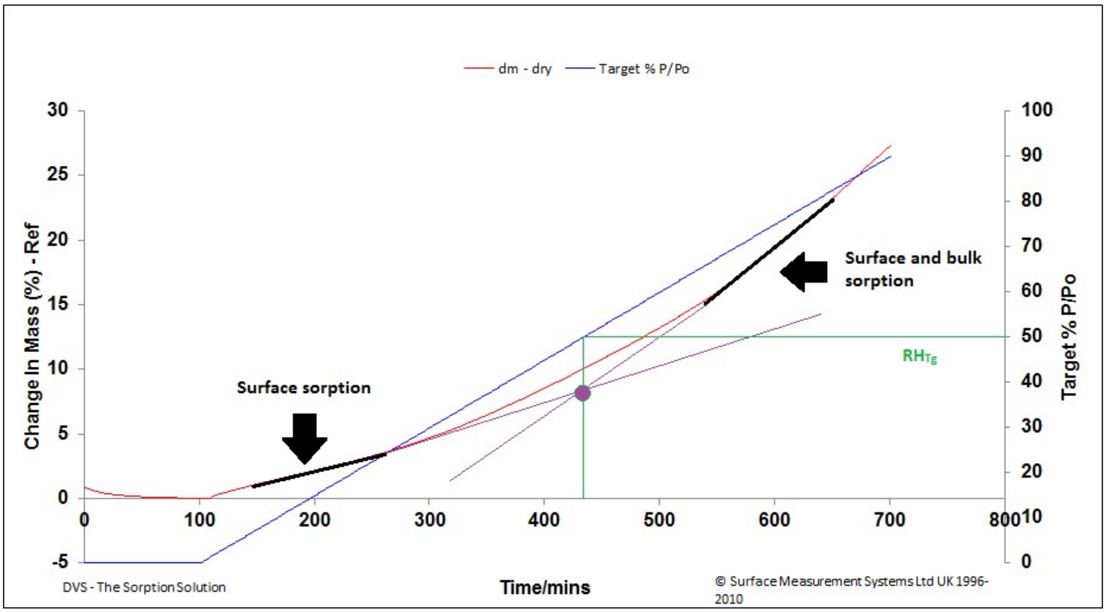

Figure S1. Sample plot of RH_{Tg} determination for melt-quenched cryo-milled PVP30KETO system.

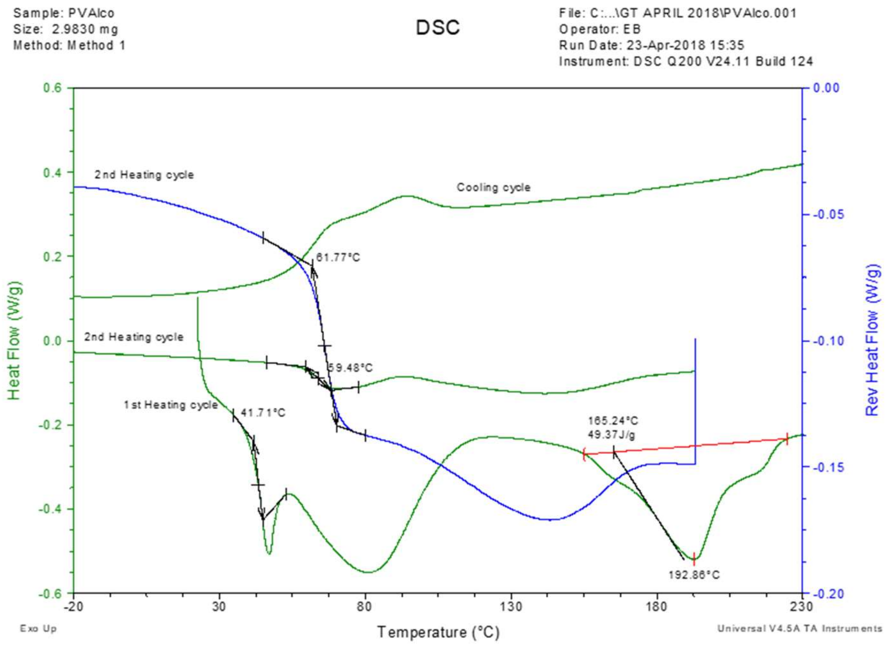

Figure S2. Differential scanning calorimetry scans showing glass transitions/melt endotherm of PVAlcohol.

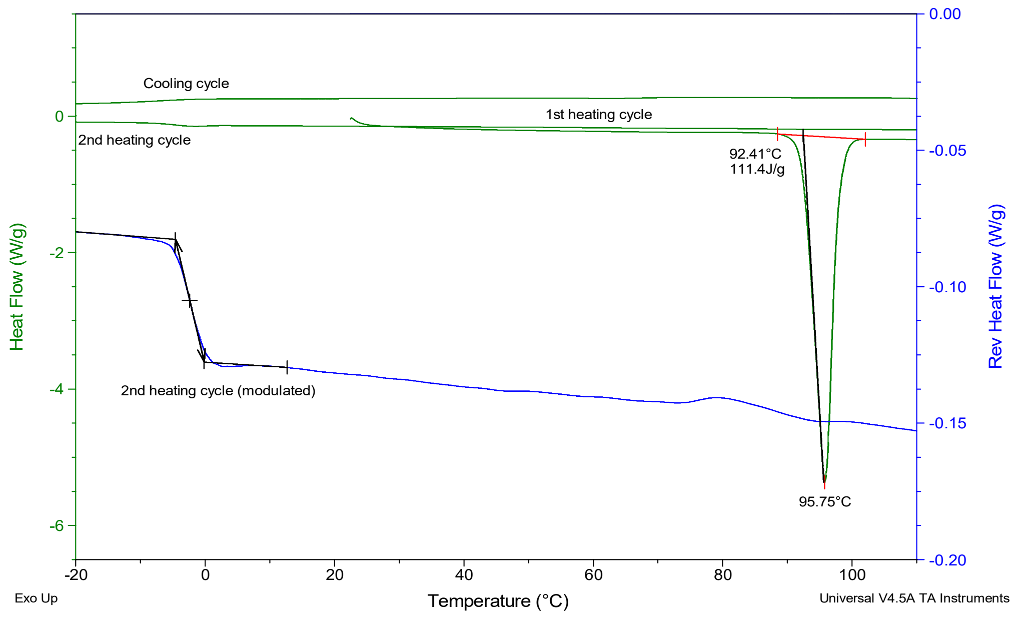

Figure S3. mDSC calorimetry scans showing melt endotherm and glass transition of ketoprofen.

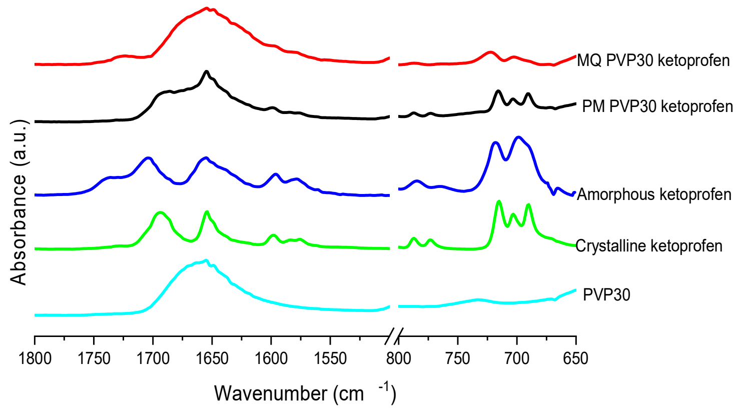

Figure S4. PVP30 and ketoprofen ATR-FTIR spectra. Melt-quenched (MQ) PVP30KETO and physical mixture (PM) PVP30KETO.

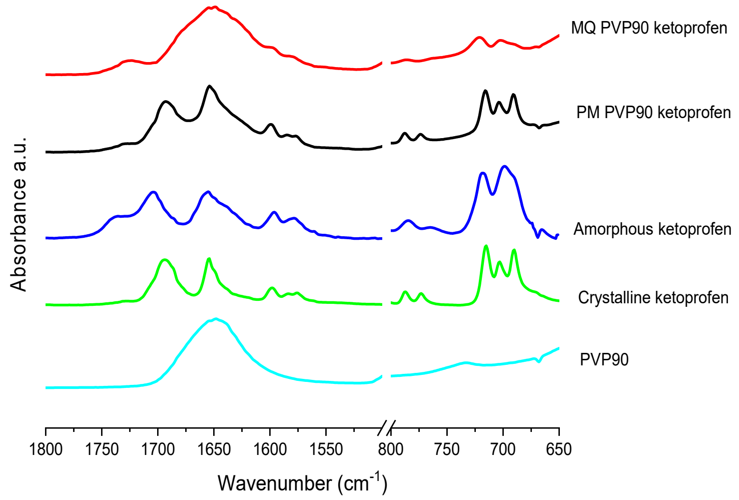

Figure S5. PVP90 and ketoprofen ATR-FTIR spectra. Melt-quenched (MQ) PVP90KETO and physical mixture (PM) PVP90KETO.

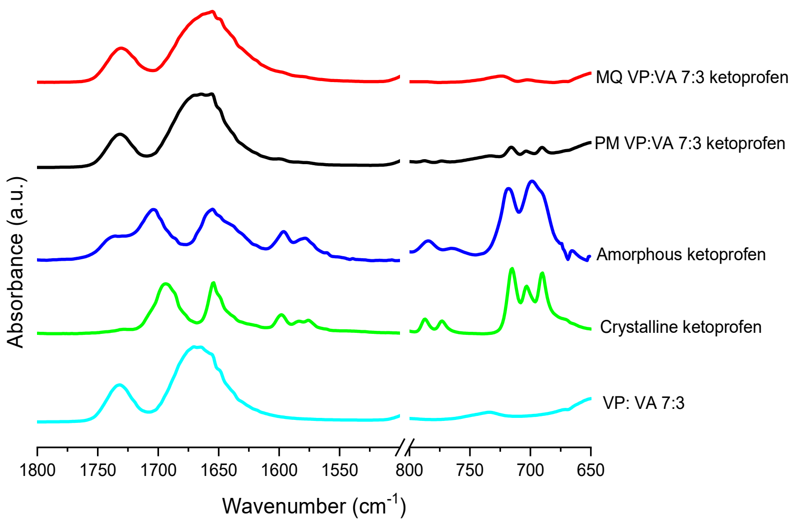

Figure S6. PVP:VA 7:3 and ketoprofen ATR-FTIR spectra. Melt-quenched (MQ) VP:VA 7:3 KETO and physical mixture (PM) VP:VA 7:3 KETO.

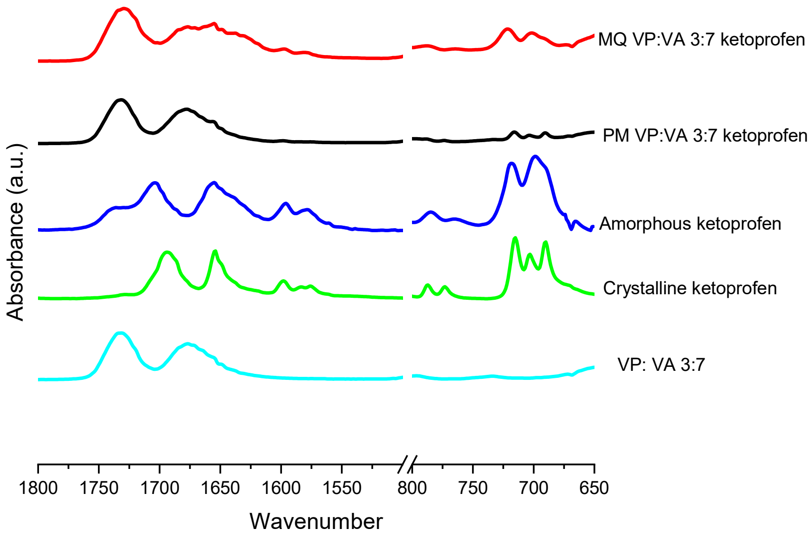

Figure S7. PVP:VA 3:7 and ketoprofen ATR-FTIR spectra. Melt-quenched (MQ) VP:VA 3:7 KETO and physical mixture (PM) VP:VA 3:7 KETO.

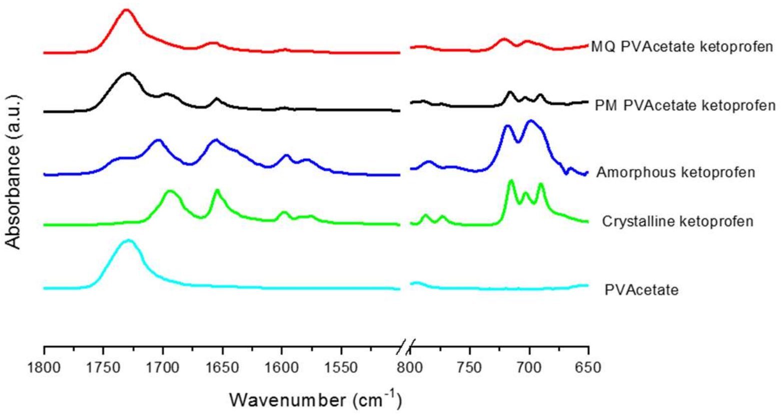

Figure S8. PVAcetate and ketoprofen ATR-FTIR spectra. Melt-quenched (MQ) PVAcetate KETO and physical mixture (PM) PVAcetate KETO.

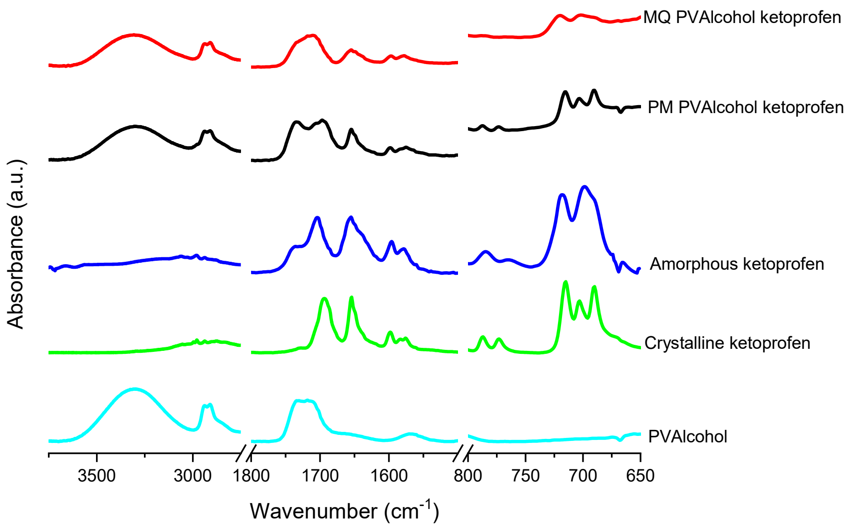

Figure S9. PVAlcohol and ketoprofen ATR-FTIR spectra. Melt-quenched (MQ) PVAlcohol KETO and physical mixture (PM) PVAlcohol KETO.

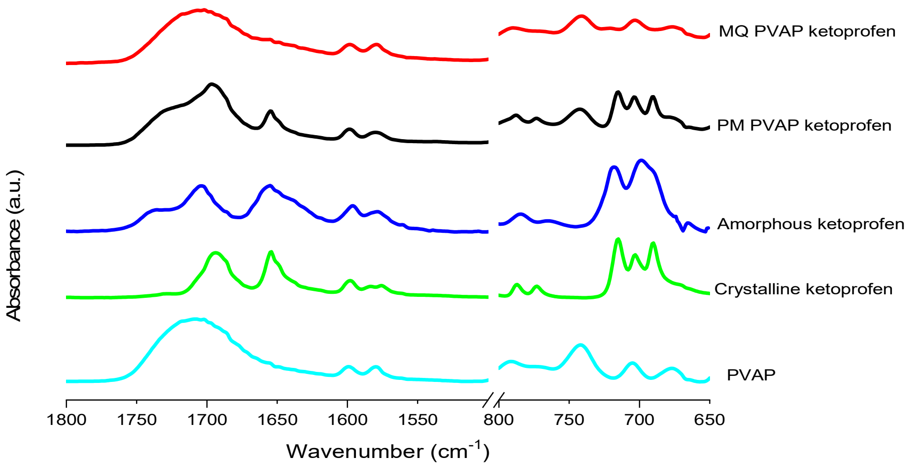

Figure S10. PVAP and ketoprofen ATR-FTIR spectra. Melt-quenched (MQ) PVAP KETO and physical mixture (PM) PVAP KETO.

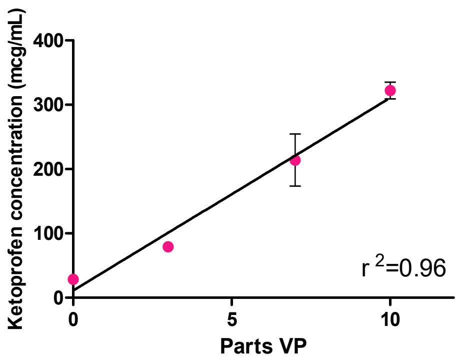

Figure S11. Dynamic solubility of vinyl pyrrolidone KETO systems at pH 1.2 at 2 hours versus parts vinyl pyrrolidone in the polymer.

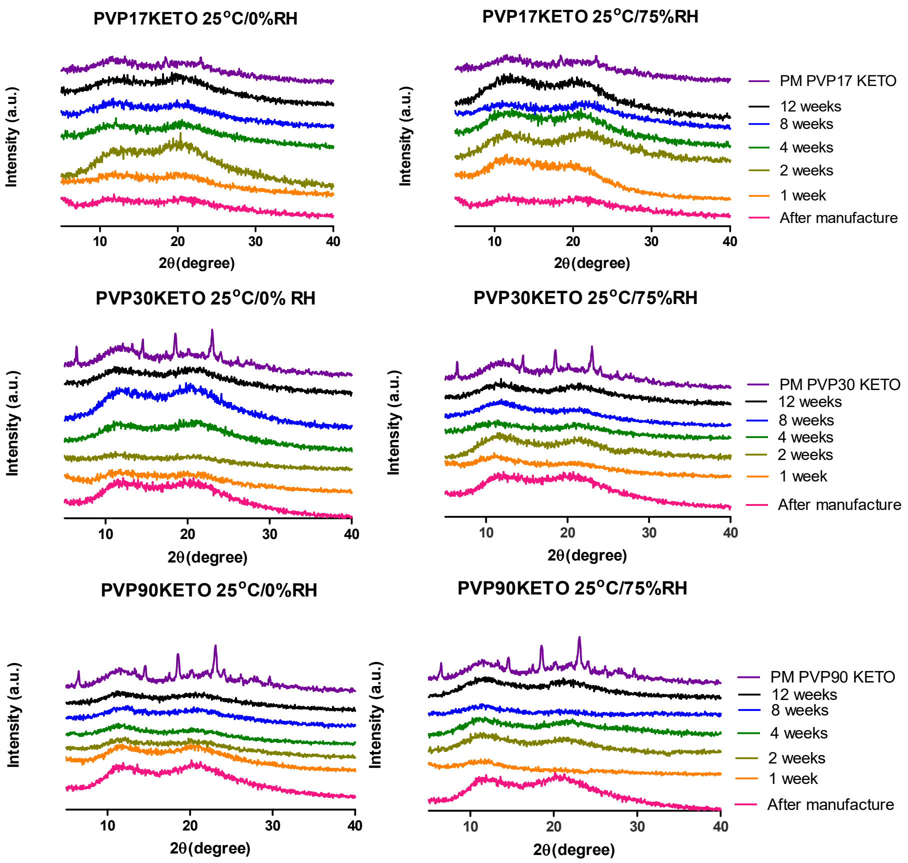
PVP17KETO 25°C/0%RH
PVP17KETO 25°C/75%RH
PM PVP17 KETO
12 weeks
8 weeks
4 weeks
2 weeks
1 week
After manufacture
PVP30KETO 25°C/0% RH
PVP30KETO 25°C/75%RH
PM PVP30 KETO
PVP90KETO 25°C/0%RH
PVP90KETO 25°C/75%RH
PM PVP90 KETO
Intensity (a.u.)
2θ(degree)
10
20
30
40

(a)

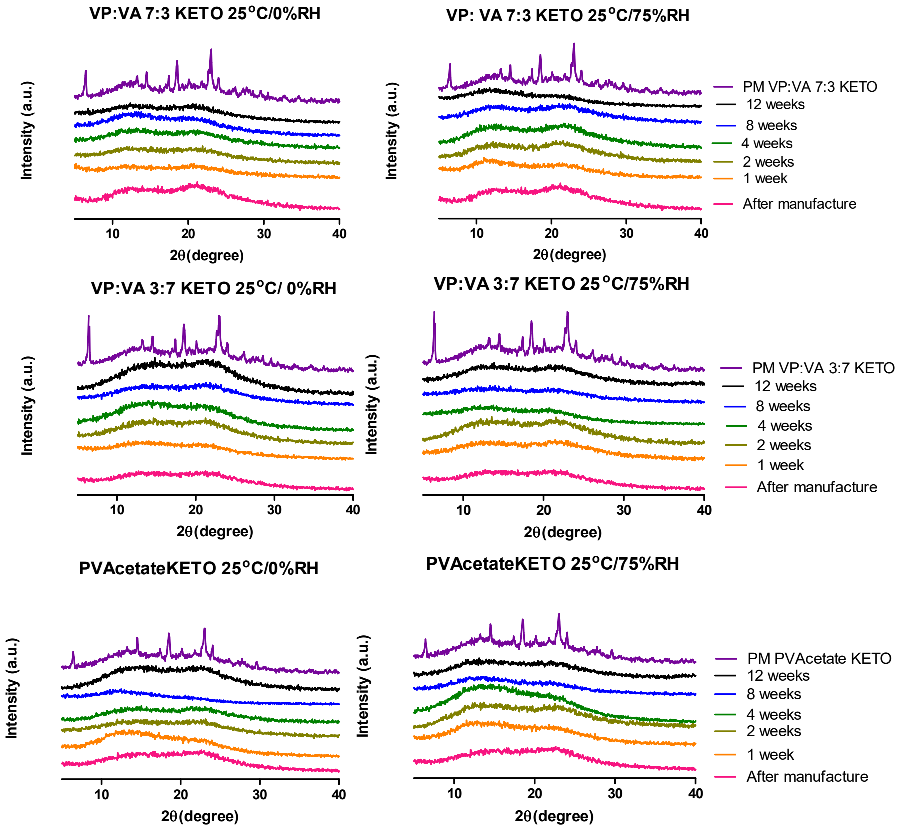
VP:VA 7:3 KETO 25°C/0%RH
VP: VA 7:3 KETO 25°C/75%RH
PM VP:VA 7:3 KETO
12 weeks
8 weeks
4 weeks
2 weeks
1 week
After manufacture
VP:VA 3:7 KETO 25°C/ 0%RH
VP:VA 3:7 KETO 25°C/75%RH
PM VP:VA 3:7 KETO
PVAcetateKETO 25°C/0%RH
PVAcetateKETO 25°C/75%RH
PM PVAcetate KETO
Intensity (a.u.)
2θ(degree)
10
20
30
40

(b)

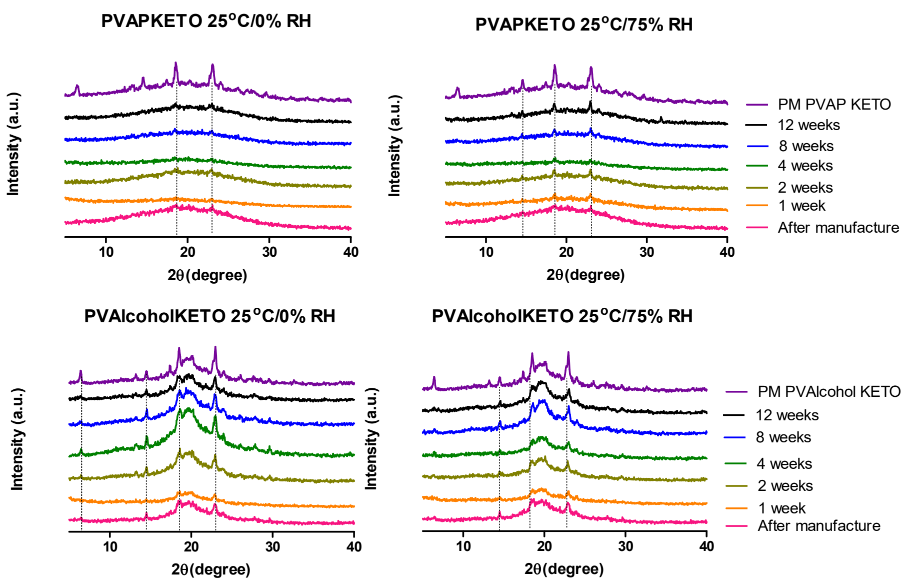

(c)

Figure S12. a). pXRD patterns of PVPKETO systems stored at 0% or 75% RH at 25 °C for 12 weeks. **b).** pXRD patterns of VP: VA 7:3 KETO, VP: VA 3:7 KETO and PVAcetateKETO systems stored at 0% or 75% RH at 25 °C for 12 weeks. **c)** pXRD patterns of PVAPKETO and PVAlcoholKETO systems stored at 0% or 75% RH at 25 °C for 12 weeks.

Table S1. Gordon Taylor predicted glass transition onset temperatures and experimental glass transition onset and offset temperatures and glass transition temperature widths for POLYMERKETO systems with 20% *w/w* ketoprofen. Values are presented as an average ± standard deviation.

**Material**	**Gordon Taylor predicted Tg onset (° C)**	**Experimental Tg onset (° C)**	**Deviation from Gordon Taylor Tg onset (° C)**	**Experimental Tg offset (° C)**	**Experimental Tg widths (°C)**
PVP 17 KETO	94.35	63.28 ± 0.95	−31.08	80.48 ± 1.87	17.20 ± 1.97
PVP 30 KETO	106.67	61.66 ± 3.78	−45.02	88.58 ± 1.32	26.91 ± 2.47
PVP 90 KETO	117.50	49.44 ± 1.59	−68.06	74.85 ± 6.24	25.41 ± 5.56
VP:VA 7:3 KETO	80.16	58.25 ± 4.83	−21.92	88.95 ± 2.17	30.70 ± 3.32
VP:VA 3:7 KETO	40.68	36.01 ± 2.52	−4.67	54.68 ± 1.36	18.67 ± 2.23
PVAcetate KETO	29.35	21.61 ± 1.97	−7.74	31.63 ± 1.79	10.02 ± 0.53
PVAlcohol KETO	29.90	27.47 ± 1.96	−2.44	65.94 ± 8.25	38.47 ± 9.22
PVAP KETO	79.67	51.39 ± 1.26	−28.28	79.79 ± 5.40	28.41 ± 6.47
